# Is Orthodontic Treatment with Microperforations Worth It? A Scoping Review

**DOI:** 10.3390/children9020208

**Published:** 2022-02-06

**Authors:** Cinzia Maspero, Annalisa Cappella, Claudia Dolci, Maria Grazia Cagetti, Francesco Inchingolo, Chiarella Sforza

**Affiliations:** 1 Department of Biomedical, Surgical and Dental Sciences, School of Dentistry, University of Milan, 20122 Milan, Italy; cinzia.maspero@unimi.it (C.M.); mariagrazia.cagetti@unimi.it (M.G.C.); 2 Fondazione IRCCS Cà Granda, Ospedale Maggiore Policlinico, 20122 Milan, Italy; 3 Department of Biomedical Sciences for Health, Università degli Studi di Milano, 20133 Milano, Italy; annalisa.cappella@unimi.it (A.C.); claudia.dolci@unimi.it (C.D.); 4 U.O. Laboratorio di Morfologia Umana Applicata, IRCCS Policlinico San Donato, 20097 San Donato Milanese, Italy; 5 Department of Interdisciplinary Medicine, Università degli Studi di Bari “Aldo Moro”, 70124 Bari, Italy; f.inchingolo@icloud.com

**Keywords:** orthodontic treatment, micro-osteoperforations, split mouth, RCTs, PRISMA Guidelines

## Abstract

Malformations of teeth and dental arches can produce functional modifications intermingled with esthetic alterations. Children’s rehabilitation may be long, requiring multiple interventions. One of the main challenges of contemporary orthodontics is to reduce treatment time by accelerating orthodontic tooth movements. Among the currently used methods, micro-osteoperforations (MOPs) are flapless, minimally invasive perforations that induce a local trauma to the bone, increase healing capacity, and accelerate dental movements. The use of MOPs in orthodontics is spreading but there are no definite and recognized protocols for their application. This scoping review collected the available evidence in the effect of MOPs during orthodontic therapy as compared to current treatments, to summarize the evidence. The guidelines proposed by PRISMA-ScR were followed: original clinical studies carried out from 2010 to 2021 were retrieved by medical databases combining the terms “micro-osteoperforations” and “accelerated orthodontic tooth movement”. From a total of 965 articles, nine were finally selected. The studies’ aims, designs, methods, measurements, outcomes, and main findings were very heterogenous, with a duration ranging from 4 weeks to 7 months. This included only Class I malocclusion to any malocclusion. It assessed the effects of MOPs coupled with a variety of orthodontic mechanics on either the retraction of maxillary canines, the distalization of maxillary molars, or the modifications on premolar roots. Mostly, variations in the number, location, and timing of MOPs impeded a global assessment. Overall, most of the studies (six out of nine) reported moderately useful effects of MOPs, one was negative, and only two found significant advantages of MOPs over conventional treatment. The review synthesized the available evidence about MOP applications in orthodontics and identified some important gaps in knowledge that could be starting points for a systematic review of the literature. In conclusion, even if MOPs can accelerate tooth movements, the variety of aims and methods of the published research prevents suggestion of their widespread use.

## 1. Introduction

Variations in size, shape, reciprocal positions, and relationships of teeth and dental arches are among the most common abnormalities in humans. These include a variety of presentations ranging from mild to severe, involving a single tooth or the whole dental arch, a genetic or acquired cause, and isolated or associated with other pathologies. Malformations and malocclusions can produce various disorders, and functional modifications are intermingled with esthetic alterations [1,2,3]. Depending on the severity of the case, children’s rehabilitation may be long, beginning in early childhood and continuing during adolescence and young adulthood, often requiring multiple interventions. The final burden on patients, families, and oral health professionals may be very distressing.

Indeed, one of the main challenges of contemporary orthodontics is to reduce treatment time. A prolonged treatment is a negative factor that can badly influence orthodontic therapy in terms of compliance and frequency of adverse effects. In particular, the prolonged duration affects the psychological state of patients who expect a much shorter duration of the treatment, thus losing confidence in its effectiveness. It is also associated with other negative sequelae [4,5,6]. Possible complications such as root resorption, discomfort, and pain, as well as bacterial time-load factors, such as white spot lesions, dental caries, gingival, and periodontal diseases, may be associated with prolonged times for the therapy [4,7,8].

Fixed appliances therapy to correct malocclusions generally requires an average treatment time of 19.9–24 months [7,9]. Complex orthodontic cases may need longer treatment duration and/ or multiple interventions, hence exposing to additional risk factors including demotivation of patients, parents, and orthodontists in addition to increased costs [10,11]. Therefore, accelerating orthodontic tooth movement (OTM) could help in shortening the treatment time. It may also prevent, reduce, or eliminate side effects and, consequently, increase satisfaction [5].

Researchers have endeavored to identify different methods to enhance the rate of OTM [9]. It is possible to classify the procedures aimed to reduce treatment time into three major categories: biologic, with the administration of local or systemic drugs; mechanical or physical, with the use of vibration and low-energy lasers; and surgical. The latter includes dentoalveolar distraction, alveolar surgeries to undermine interseptal bone, micro-osteoperforations (MOPs), and alveolar corticotomies [7].

Corticotomy is the most popular surgical method. It has the largest amount of research evidence supporting its efficacy in OTM speed-up owing to regional acceleratory phenomena [12]. Namely, tissue reaction to any noxious stimulus that increases the healing capacity is seen in both hard and soft tissues of the oral cavity [13].

Tissue response is characterized by increased perfusion, bone turnover, and decreased bone density, all caused by the generation of inflammatory markers described below [14]. According to Frost, a repairing tissue recovers faster if it undergoes a regional stimulant intervention [5]. Hence, inducing trauma to the bone in the region in which acceleration is required, can consequently accelerate the OTM [13]. Nonetheless, an extensive surgery such as a corticotomy would likely discourage the patient [7]. Although proven to be successful, patients are unwilling to sustain corticotomies to reduce orthodontic treatment duration [12].

Since invasive surgical insult to the bone, periosteum, and mucosa are unpleasant for the patients, in recent years the traditional surgical methods have been replaced by minimally invasive techniques avoiding the elevation of mucoperiosteal flaps [9]. Among them, MOPs or alveocentesis, practiced since 2010, are among the least invasive surgical procedures used to accelerate orthodontic treatment. A set of transmucosal holes are made in the cortical bone of the selected part of the oral cavity using mini-screws; it is not needed to raise a mucoperiosteal flap. Therefore, the integrity and architecture of the hard and soft tissues are mostly maintained [4,14,15].

Considering these favorable properties, the use of MOPs in orthodontics is spreading. However, there seem to be no definite and recognized protocols for their application and the quantitative assessment of their effects and limitations as compared to classic treatments. Therefore, it seems necessary to verify the relevant literature, including how research is conducted, in order to synthesize the current evidence [16]. This scoping review aims to share existing available evidence in the effect of MOPs during orthodontic therapy as compared to current treatments to systematically map the research conducted in this area as well as to identify any existing gap in knowledge. The results may support the need for a new updated systematic review of the literature.

## 2. Materials and Methods

Guidelines proposed by PRISMA-ScR (Preferred Reporting Items for Systematic reviews and Meta-Analyses extension for Scoping Reviews) [16,17,18] were followed to conduct this scoping review. The main medical databases were consulted (PubMed, Embase, Scopus, Google Scholar) to seek clinical studies carried out from 2010 to 2021 The search strategy combined the terms “micro-osteoperforations” and “accelerated orthodontic tooth movement” as keywords, using Boolean operators “AND” and “OR” to select original papers focused on MOPs and orthodontic treatment. The field of research was restricted to articles exclusively in English and concerning humans. The identified publications were read and independently evaluated by two reviewers considering the eligibility criteria. Divergences were resolved after consensus by both reviewers. To summarize, the following inclusion criteria were considered:

- Studies in the English language;
- Studies from 2010 to date;
- Studies involving humans;
- Studies evaluating tooth movement in association with MOPs;
- Randomized clinical trials (RCTs);
- Prospective studies.

Whereas these exclusion criteria were followed:

- Case reports;
- Systematic reviews and meta-analyses;
- Narrative reviews;
- Studies that evaluated accelerated tooth movement by other methods (i.e., piezocision, low-frequency mechanical vibrations, photobiomodulation therapy, and local injection of platelet-rich plasma).

## 3. Results

**Figure 1:**
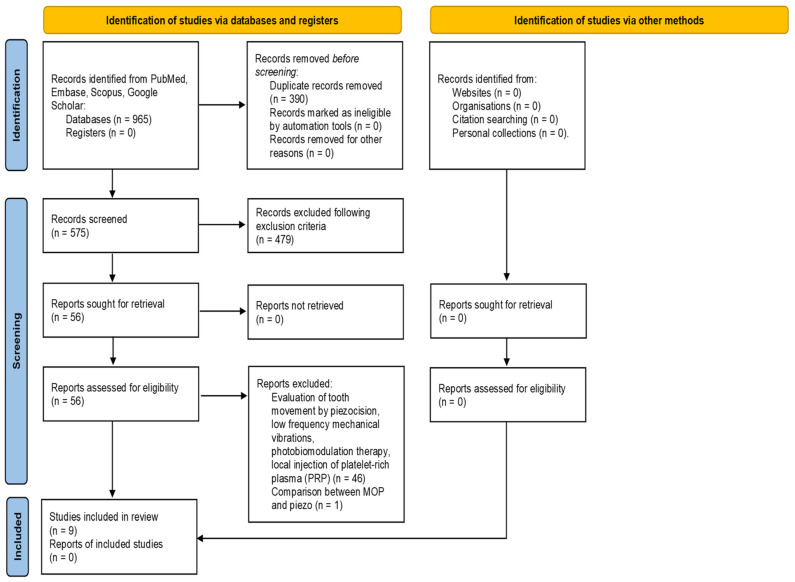
PRISMA 2020 flow diagram for new systematic reviews, which included searches of databases, registers, and other sources.

A total of 965 studies were identified from searches of electronic databases and review articles references. After removing duplicates, 575 articles were found. Subsequently the research was further restricted by applying a temporal filter. A total of 479 articles published in the last 12 years were selected. Among them, 249 articles were performed on humans, and only 55 studies were RCTs and prospective studies. Based on the title and abstract, 45 of them were excluded because techniques different by MOPs were used to increase the rate of OTM. Afterwards, of 10 articles analyzed, another one was excluded because it compared the effects of MOPs vs piezocision in accelerating OTM in adults. Finally, a total of nine articles published between 2013 and 2020 were evaluated in the present research (Figure 1). The studies’ aims, designs, methods, measurements, outcomes, and main findings are summarized in Table 1.

### 3.1. Study Design

Randomized clinical trials were selected for this scoping review [4,5,6,12,13,15,20,21] except for the prospective conducted by Chan et al. [14]. Among them, seven studies had a split-mouth design [4,5,12,14,15,20,21] and the remaining used a parallel-arm design [6,13].

### 3.2. Study Duration

No homogeneity was found regarding the duration of studies. Four studies concluded after 4 weeks of observation [6,13,14,21], two papers after 12 weeks [4,5], another two studies after 16 weeks [12,15], and only one study lasted 7 months (from December 2018 to July 2019) [20].

### 3.3. Population

Three studies included only molar class II malocclusion patients [4,5,21], Babanouri et al. [20] included bilateral class II division 1 malocclusions or class I malocclusion with bimaxillary protrusion patients. In the remaining ones, no information about the skeletal or dental class was reported, and patients with any malocclusion were included as long as including the second permanent molars was required [6,12,13,14].

### 3.4. Intervention

To investigate the effects of MOPs on the rate of tooth movement, seven studies evaluated the retraction of maxillary canines [4,6,12,13,15,20,21]. Instead, Gulduren et al. considered the distalization of maxillary molars [5], while Chan et al. [21] focused on root resorption of maxillary premolars in a group of patients who required orthodontic treatment with premolars extraction.

In all studies but one [12], patients of both sexes were enrolled. Most of the patients were late adolescents or young adults [4,5,12,13,15,20,21]; in two studies the mean age of participants was 15 years [6,14]. Indeed, the inclusion criteria of most studies included adolescents [4,5,6,20], starting from 12 [14] or 13 years of age [13]. In four studies, permanent dentition premolars observed root resorption after MOP [14]. All studies included the extraction of dental elements except Gulduren et al. [5]; extracted teeth were the upper first molars [4,21], the first maxillary premolars [6,12,14,20], or all four first premolars [13,15].

### 3.5. Mechanics for Treatment

In the study proposed by Gulduren et al. [5], the effects of MOPs on the rate of molar distalization supported by mini-screws were studied. Two mini-screws were inserted at the level of the anterior palate and an individually designed distalization mechanics was built. MOPs were performed on the first day of the distalization treatment and repeated every 3 weeks for three times (six MOPs each time). Chan et al. [14] studied root resorption of extracted teeth in the proximity of MOPs. Bioquick brackets bonded on 16/26 and 14/24 and cantilever springs applied a buccally directed force to the maxillary first premolars. MOPs were performed on both the mesial and distal aspects of the selected first premolar in the buccal alveolar region, and the teeth were extracted 4 weeks after the MOPs.

In the remaining seven articles, the effects of MOPs on canine retraction were investigated [4,6,12,13,15,20,21]. In five investigations, closed-coil nickel-titanium springs were used, but with different methods to move the canine tooth into the arch. Alkebsi et al. [4] used mini-screws. Alikhani et al. [21] applied the force closer to the center of resistance of the tooth, while Babanouri et al. [20] used a power arm on the canine surface to induce bodily movement and placed 1.6 mm diameter mini-screws between the roots of the second premolar and the first permanent molar. NiTi closing coil springs were used also by Aboalnaga et al. [12] and Haliloglu-Ozkan et al. [6].

Sivarajan et al. [15] retracted the canines on the working archwire using an elastomeric chain force. Attri et al. [13] proposed canine retraction using second molar banding and a transpalatal arch.

### 3.6. MOPs Applications

Six out of nine studies used orthodontic mini-screws to perform MOPs [4,6,8,12,15,20], while a special tool, the PROPEL device, was used in the remaining three [13,14,21]. The PROPEL device is a stainless-steel screw attached to a dedicated motor designed for this purpose [5,13,21].

In all studies [4,6,12,13,15,20,21], three MOPs were performed, except for Gulduren et al. [5] and Chan et al. [14]. In the latter, only two MOPs were performed, mesially and distally to the first maxillary premolar to be extracted. In the former, MOPs were performed at T0 and then every 3 weeks. For each session, two MOPs were performed between the second premolar and the first upper molar, two more between the first molar and the second molar, and the last two distal to the second molar, for a total of six MOPs.

While two studies [12,21] gave no indications regarding how MOPs should be performed, in the others MOPs were performed at different sites and with a variable distance from each other. For example, in the study by Alkebsi et al. [4], the MOPs were performed 3 mm away from the canine and 6 mm away from the gingival margin. The remaining MOPs were performed 5 mm away from the former. Sivarajan et al. [15] performed MOPs at 2 mm distance from each other. Attri et al. [13] performed three MOPs at 1.5 mm intervals, Haliloglu-Ozkan et al. [6] performed them as close as possible to the canine apex by doing the MOPs vertically. In Babanouri et al. [20] study, three MOPs were performed on the buccal surface of the alveolar process on the experimental side of the MOP1 group while, in the MOP2 group, three MOPs on the buccal surface and three MOPs on the palatal surface were created. MOPs were performed in the middle of the distance between the distal surface of the canine and the mesial surface of the second premolar. The first MOP was located 5 mm away from the free gingival margin. Gulduren et al. [5] performed MOPs in the maxillary molar buccal alveolar regions, while Chan et al. [14] made them in the mesial and distal aspects of the buccal alveolar region of the first premolar.

### 3.7. Synthesis of Results

The nine studies that this scoping review was based on considered different patients, methods, timing, devices, and modality of research outcome assessment, making a synthesis very difficult. Overall, most of the investigations reported that MOPs were potentially useful and may help accelerate orthodontic treatments. Differences in space closure and canine retraction, up to 2.3 times faster in the treated side than in the control side, were reported [13,21].

Chan et al. [14] demonstrated that MOPs cause greater root resorption than the control group. Five studies [6,13,15,20,21] stated that MOPs were useful in increasing the rate of tooth movement. In contrast, Gulduren et al. [5] asserted that the accelerating effect of MOPs was less than expected, and other studies [15,20] stated that the statistically significant increment in dental movements was not clinically useful. Alkebsi et al. [4] and Aboalnaga et al. [12] stated that MOPs were not able to accelerate the rate of canine retraction; however, they seemed to facilitate root movement.

Overall, most of the studies (six out of nine) reported moderately useful effects of MOPs [5,12,13,14,15,20], one was negative [4], and only two investigations found significant advantages of MOPs over conventional treatment [6,21].

## 4. Discussion

According to epidemiological surveys performed in Italy and India (Chennai state), the percentage of children and adolescents who do not receive the necessary dental health care may be even higher than 50%. Interceptive, early orthodontic treatment may help in reducing this percentage [1,2]. From this point of view, the use of minimally invasive surgical interventions may play an important role.

In the current scoping review, we summarized the up-to-date evidence about the effects of a surgical technique that can help in reducing the duration of orthodontic treatments with limited side effects. The procedure was developed and described for orthopedic treatments outside the oral cavity, and its efficacy was explained by regional acceleratory phenomena in the nearby cells [23].

MOPs are minimal perforation of cortical bone conducted using instruments such as mini-screws without the necessity to raise a flap. The minimally invasive technique produces only local, small damages to the mucosa, which maintains its integrity and architecture [14].

The multiple transmucosal perforations are performed in the proximity to the area where OTM is desired, with specific configurations depending on the required tooth movement [15]. The induced movement depends on bone resorption produced by the osteoclasts; increasing their activity should accelerate tooth movement. Indeed, performing MOPs during OTM can induce the production of inflammatory markers, such as chemokines and cytokines (TNF α, IL-1, and IL-6 locally) [24]. Chemokines play an important role in the recruitment of osteoclast precursor cells, while cytokines lead the differentiation of osteoclasts from their precursor cells to mature ones. High osteoclasts activity can result in a major rate of tooth movement [13,21].

In addition to osteoclasts activity, the increased apoptosis and proliferation of periodontal ligament cells may play an important role in accelerating tooth movement when MOPs are performed, as reported by a study conducted in animals [24].

Most of the problems associated with conventional surgical methods could be managed with MOPs. MOPs can be performed directly by the orthodontist without the collaboration of another specialist, using common orthodontic devices. Therefore, it is possible to increase or simplify the OTM and adjust the anchorage, reducing the overall healthcare cost for the patient [8,13].

Many human clinical trials that experimented with MOPs, during orthodontic treatment as compared to standard techniques, reported an increased OTM [25] with a reduction of treatment time between approximately 30% [15] and 62% [21]. They also underlined the minimal discomfort reported by the patients, [4,13,15,21] with only mild pain, and eating and speech difficulty, in the short term [5]. Pain perception was investigated by Attri et al. [13] 1, 7, and 28 days after MOPs, and no statistically significant differences were found. The only mild discomfort described was perceived 24 h after the intervention and it gradually faded away [13]. In another study [12], the pain rate experienced ranged from mild to moderate, and it rapidly disappeared after 1 week.

Overall, it seems that MOPs can be performed with no distress for the patient [21]. The use of a topic anesthetic can help reduce the mild discomfort caused by the injections needed for the technique.

The effect of MOPs on root resorption, observed as a side effect of prolonged orthodontic therapies, is still controversial [19,25]. Both no differences with the control group [4,12], and a higher amount of root resorption in cases treated with MOPs, were reported [8,14].

Controversial results were reported in the literature about the efficiency of MOPs [8,19,22]. In recent studies by Alkebsi et al. [4] and Aboalnaga et al. [12], contradictory findings were described regarding the rate of tooth movement between MOP and control groups. The authors claimed that this technique was inadequate to activate a regional inflammatory response that accelerated OTM. The same results were recently reported by Bolat Gümüş and Kınsız [22], who could not find any significant effect of MOPs on the rate of OTM in a period of three months. By superimposing baseline 3D digital imaging of canine retraction on the 3D digital image [12] of study models that were taken monthly for the first three months of treatment, they concluded that MOPs using temporary anchorage devices did not accelerate the rate of OTM.

Halioglu et al. [6] measured the effects of two repetitions of MOPs executed at 4 weeks. They noted anchorage canine loss during the retraction treatment without important difference between the treated and control groups; in all patients, the anchorage was reinforced with temporary anchorage devices inserted mesial to the first molars. The authors concluded that MOPs cause neither augmented nor anchorage loss, as previously described by Shahabee et al. [8].

No direct assessments of the effect of age on orthodontic treatments including MOPs were reported [19], even when patients younger than 16 years seemed to present a better response to treatment [5]. Indeed, among the analyzed investigations, two studies were performed on patients with a mean age of 15 years, and both reported a significant effect of MOPs [6,14].

One of the limitations of our scoping review was the language restriction: only English-language publications were included in the search, thus all clinical trials published in other languages were neither included nor taken into consideration. In addition, we employed a thorough search of the published literature while neither the grey literature nor study protocols were searched, which may have prevented the consideration of additional important findings.

Moreover, this scoping review highlighted the great heterogeneity still existing among all the studies, in terms of study design, sample size and age, methodological appraisal of main outcomes, and follow-up treatment. Hence, the differences in their results may depend on this heterogeneity, since all studies investigated the effect of MOPs for a short evaluation period. The effectiveness of the treatment was only reported based on partial evaluations, a limitation that highlights the need for additional and more consistent clinical trials. Furthermore, no short-, mid-, or long-term assessments of relapse, dental arch stability, and side effects were performed.

## 5. Conclusions

Overall, considerable methodological heterogeneity of the studies selected for this scoping review can be highlighted. The studies differed for their use of surgical techniques, the mechanism of measured OTM, the method of measurement, and the reference points considered. Only two investigations were fully positive about the effects of the surgical treatment, another six were moderately positive, and one was negative.

Literature regarding the effectiveness of MOPs in accelerating OTM is not conclusive for several reasons. The short duration of the studies and the great heterogeneity of the available evidence are the principal reasons. Moreover, some of the papers have a reduced sample size, combine patients with a variety of treatment needs, and combine a wide age range. In addition, the research on MOPs is mainly limited to canine movements while the other teeth are less considered. Literature needs more studies about the increase in parameters of periodontal disease and root resorption during and after this mini-invasive, surgical procedure.

The current scoping review synthesized the available evidence about MOP applications in orthodontics. It identified some important gaps in knowledge that could be starting points for a systematic review of the literature. Further studies are needed to discover the number and frequency of MOPs necessary to gain an accelerated OTM without side effects and to clarify if a single use of MOP with a flapless corticotomy procedure is enough to increase OTM. In conclusion, it has been shown that MOPs can accelerate OTM, but the variety of aims and methods of the published research prevents suggesting their widespread use to shorten the therapies and offer the necessary dental healthcare to a larger number of children and adolescents.

## Figures and Tables

**Table 1:** Summary of the studies’ aims, designs, methods, measurements, outcomes, and main findings.

Authors and Journal	Design	Population	Sample	Study Duration	Intervention	Results	Conclusion
Alikhani et al. [19] Am J Orthod Dentofacial Orthop 2013	RCT split mouth only for test group	Class II div. I malocclusion	10 MOPs 10; NO MOPs 20 patients; experimental group: 5 F, 5 M, mean age 27 y; control group: 7 F, 3 M, mean age 25 y	4 weeks	Canine retraction using NiTi closing coil springs anchored to a power arm on the canine bracket (Propel device used to perform MOPs). Upper molars extraction,	On average, MOPs increased the rate of canine retraction by 2.3-fold when compared with the control group	Performing MOPs is an effective, comfortable, and safe procedure to accelerate tooth movement and significantly reduce the duration of orthodontic treatment.
Alkebsi et al. [4] Am J Orthod Dentofacial Orthop 2018	RCT split mouth	Class II div. I malocclusion	16 MOP left side; 16 MOP right side 32 patients, 24 F, 8 M, mean age 19 y; inclusion > 15 y	12 weeks	Canine retraction (supported by mini-screw and closed-coil NiTi springs); Upper first molars extraction.	No significant difference in the rates of tooth movement between the MOP and the control sides mean difference, 0.2 mm; 95% CI, −0.13, 0.18 mm	MOPs were not effective in accelerating tooth movement
Sivarajan et al. [20] Angle Orthod 2018	RCT split mouth (MOP1 vs MOP2 vs MOP3 group)	Class I; Class II, Class III malocclusions	10 MOP 4-weekly maxilla8-weekly mandible; 10 MOP 8-weekly maxilla 12-weekly mandible; 10 MOP 12-weekly maxilla 4-weekly mandible 30 patients, 23 F, 7 M, mean age 22 y; inclusion > 18 y	16 weeks	Canine retraction (supported by mini-screw on the working archwire using an elastomeric chain. Upper and lower first premolars extraction.	MOP side had a significant increased canine retraction of 1.1 mm	The increased canine retraction is unlikely to be clinically significant.
Attri et al. [18] J Orthod 2018	RCT parallel between patients	NA	30 MOP associated with multibrackets; 30 only with multibrackets; 33 F, 27 M (inclusion criteria: 13–20 y, permanent dentition). Control group: 15 F, 15 M (mean age 18 y). Experimental group (MOP): 18 F, 12 M (mean age 18 y)	4 weeks	Canine retraction using second molar banding with transpalatal arch and a tie back elastic (Propel device used to perform MOPs) Upper and lower first premolars extraction.	Mean differences in the monthly rate of space closure ranged between 0.24 and 0.37	MOPs seem to improve the rate of tooth movement without differences in pain perception
Haliloglu-Ozkan et al. [6] J Clin Exp Dent 2018	RCT parallel between patients	Class I; Class II, Class III malocclusions	18 MOP for canine retraction; 18 conventional mechanics for canine retraction, 4 lost. Experimental group: 7 F, 10 M, mean age 15 y; Control group: 6 F, 9 M, mean age 16 y, inclusion 16–25 y	4 weeks	Canine retraction (supported by mini-screw). NiTi closing coil spring. Upper first premolars extraction.	Canine distalization; mean significant differences control vs MOP 0.19, 0.4 mm	Performing MOPs is an effective method for increasing the rate of tooth movement in the maxilla.
Chan et al. [21] Am J Orthod Dentofacial Orthop 2018	Prospective controlled clinical trial, split mouth	Class I; Class II, Class III malocclusions	20 patients requiring extraction of the maxillary first premolars as part of their orthodontic treatment. 20 patients (12 F, 8 M, mean age 15 y), inclusion criteria: 12–25 y; permanent dentition	4 weeks	Extraction of first maxillary premolars to observe root resorption 4 weeks after MOP (performed by propel device, 5 mm depth). Brackets bonded on 16/26 and 14/24. Cantilever springs applied buccally directed force to 14 and 24.	Root resorption was 42% significantly larger on the MOP side than on the control one.	Performed MOPs resulted in greater orthodontic root resorption.
Aboalnaga et al. [17] Progr Orthod 2019	RCT split mouth	Class I; Class II, Class III malocclusions	18 patients requiring bilateral first premolar extraction and upper canine retraction. Before canine retraction, 3 MOPs were randomly allocated to either the right or left sides. 18 F, mean age 21 y; inclusion 16–25 y, permanent dentition	16 weeks	Canine retraction (supported by mini-screw) using NiTi closing coil springs. Upper first premolars extraction.	Mean differences -MOP vs. control- of the total distance moved by 1. The canine cusp tip 0.06 ± 0.7 mm (*p* > 0.05). 2. the canine center and apex 0.37 ± 0.63 mm (*p* < 0.05) and 0.47 ± 0.56 mm (*p* < 0.01) respectively).	Performed MOPs didn’t accelerate the rate of canine retraction; however, they seemed to facilitate root movement
Gulduren et al. [5] J Orofac Orthop 2020	RCT split mouth	Class II div. I/II malocclusions	10 MOPs to the left / right maxillary molar region; 10 no MOPs, 4 patients lost; 18 subjects (9 experimental group 4 F, 5 M, mean age 22 y; 9 control group: 3 F, 6 M, mean age 18 y, inclusion 16–24 y	12 weeks	Maxillary molar distalization. MOPs (mini-screws) at T0 of distalization treatment, repeated every 3 weeks for three times (six MOPs each time)	The molars of the MOP side more 1.17 times significantly more than the other side.	The accelerating effect of MOPs was lower than expected.
Babanouri et al. [22] Progr Orthod 2020	RCT split-mouth (MOP1 vs MOP2 group)	bilateral class IIdivision 1 malocclusions or class I malocclusion with bimaxillary protrusion	28 patients randomly allocated into two groups (MOP1 and MOP2). 3 lost. Experimental group MOP 1: 7 F, 5 M, mean age 26 y; Experimental group MOP2 7 F, 6 M; mean age 25 y. Inclusion 15–45 y	December 2018 to July 2019	Canine retraction (supported by mini-screw placed bilaterally in the buccal alveolar process). NiTi closed coil spring, temporary anchorage on the canine surface to induce bodily movements	Mean difference vs. control group (significant): MOP1 0.2 mm, MOP2 0.6 mm	MOPs interventions accelerated tooth movement and canine retraction, but the increased tooth movement following MOPs was not clinically significant.

F: female; M: male; MOP: micro-osteoperforation; NA: not available; NiTi: nickel-titanium; RCT: randomized clinical trial; y: year.

## Data Availability

Not applicable.
